# Effect of FTY720 on Some Physiological Indexes of Non-Obese Diabetic (NOD) Mice

**DOI:** 10.3390/ijms13056129

**Published:** 2012-05-18

**Authors:** Xiaoqiang Chen, Sudan Ye, Shikang Zhang, Jianrong Li, Hongying Zhu, Gaoli Zheng, Yin Lu, Haitong Wan

**Affiliations:** 1 College of Food Science and Biotechnology, Zhejiang Gongshang University, Hangzhou 310035, China; E-Mail: lijianrong@mail.zjgsu.edu.cn; 2 Hangzhou Tea Research Institute, China COOP, Hangzhou 310016, China; E-Mail: zsk651@126.com; 3 Zhejiang Economic & Trade Polytechnic, Hangzhou 310018, China; E-Mail: yesd808@163.com; 4 College of Biological and Environmental Engineering, Zhejiang Shuren University, Hangzhou 310015, China; E-Mail: zhy090909@163.com; 5 Institute of Materia Medica, Zhejiang Academy of Medical Sciences, Hangzhou 310013, China; 6 Institute of Cardio-Cerebrovascular Disease, Zhejiang Chinese Medical University, Hangzhou 310035, China

**Keywords:** FTY720, type 1 diabetes (T1D), non-obese diabetic (NOD) mice, physiological indexes

## Abstract

The studies were performed to investigate the physiological characteristics of non-obese diabetic (NOD) mice treated with FTY720. At the age of 12 weeks, each mouse was fed with FTY720 or physiological saline once a day for 10 weeks running, and their blood glucose, weight, anti-GAD antibody and organ indexes were determined. No mouse in group FTY720 (NOD mice treated with FTY720) showed diabetic symptoms. The average content of serum anti-GAD antibody in group FTY720 decreased 48.75% (*P* < 0.01). It was concluded that the spleen, kidney and liver of NOD mice treated with FTY720 shriveled significantly in the progression of diabetes (*P* < 0.01 or *P* < 0.05). The body weight of group FTY720 mice was slightly lower than that of the model control (MC) group and these two groups both had less body weight than the normal control (NC) group (*P <* 0.01). The result of tests of anti-GAD antibody suggested that FTY720 treatment could suppress the anti-GAD response.

## 1. Introduction

FTY720 (2-amino-{2-(2-[4-octylphenyl] ethyl}-1,3-propanediol hydrochloride), a novel immune regulatory drug, is a synthetic sphingosine analogue of myriocin (ISP-1) [1,2]. ISP-1 can be derived from Chinese caterpillar fungus, a complex of fungus and caterpillar which has been used for medicinal purposes for centuries, particularly in China, Korea and other Asian countries [3]. As a new class of immunosuppressants, FTY720 currently demonstrates great potential in several animal models of transplantation, including skin allograft and pancreatic islet transplantation [3–5]. FTY720 could produce a reduction of lymphocytes resulting from a redistribution of T and B lymphocytes between circulatory and secondary lymphoid tissue. The treatment of FTY720 makes T lymphocytes redistribute away from tissue grafts. That is why FTY720 could prolong transplantation survival [4–6]. Type 1 diabetes, also called juvenile diabetes or insulin-dependent diabetes mellitus (IDDM), is the consequence of progressive T cell-mediated autoimmune destruction of pancreatic β cells. The body’s immune system attacks and destroys insulin-producing pancreatic β cells in the islets of Langerhans, resulting in a lack of insulin [7,8].

FTY720 has proven effective in the prevention and cure of diabetes in non-obese diabetic (NOD) mice [2,8,9], but essential physiological characteristics, including weight, autoantigen, such as glutamic acid decarboxylase (GAD), and major organ indexes that may be affected have rarely been addressed. Many studies suggest that IDDM in NOD mice and patients is mediated by T cells. The presence of GAD-reactive T cells in T1D patients is clearly confirmed. The autoimmune responses to GAD precede in the development of T1D [7,10]. Therefore, the anti-GAD autoantibody is an important marker of T1D. The physiological characteristics are important to study toxicology research on FTY720 and likely differ from investigations of the immunological mechanism at the molecular level. For a better understanding of its biological activity, we performed a study to evaluate the treatment of FTY720 to Type 1 diabetes in non-obese diabetic (NOD) mice, which is considered the best animal model of the human disease. The result will contribute to evaluate therapeutic feasibility of FTY720 through investigating physiological characteristics of the treated NOD mice.

## 2. Results

### 2.1. The Level of Blood Glucose in NOD Mice

The results can be seen from Table 1. The level of blood glucose of group MC mice went up with the increasing age. Compared with group NC mice, that of group MC mice was significantly higher during the age of 19–22 weeks (*P <* 0.05 or *P <* 0.01). The level of blood glucose of group FTY720 mice was significantly lower than that of group MC mice since their age of 17 weeks (*P <* 0.05 or *P <* 0.01), approximates to, or is significantly lower than, that of normal control mice during the trial (*P <* 0.05 or *P <* 0.01). The results revealed that FTY720 could effectively inhibit the exaltation of blood glucose in NOD mice.

### 2.2. The Incidence of Diabetes in NOD Mice

The incidence of diabetes is shown in Table 2 as a cumulative number in each group. In the third week of the trial, diabetes appeared in 2 group MC mice. The incidence of diabetes of group MC mice had significantly risen from the 5th to 10th weeks of the trial (*P* < 0.05 or *P* < 0.01) and reached 70% at the end, whereas in the group FTY720 and group NC, no mouse showed diabetic symptoms. The result demonstrated FTY720 can effectively suppress the diabetes onset in NOD mice.

### 2.3. The Level of Anti-GAD Antibody in NOD Mice

Table 3 shows the level of anti-GAD antibody in serum of mice in the groups. Compared with anti-GAD antibody in group NC mice, the one in group MC mice was significantly very high (*P* < 0.01). Compared with model control mice, the average content of anti-GAD antibody in the serum of group FTY720 mice decreased by 48.75% and showed a very significant difference (*P* < 0.01), but significantly higher than that of normal control mice (*P* < 0.05). The autoimmune responses to GAD (GAD65) were pivotal in the development of T1D [10–12]. Many studies demonstrated that the proliferative CD4^+^ T-cell response to GAD65 (one of the pancreatic β-cell antigens) was a relevant marker for cellular autoimmunity in T1D. The lower level of serum anti-GAD antibody of NOD mice treated with FTY720 suggested that FTY720 could markedly suppress GAD-reactive T cells autoimmune responses.

### 2.4. The Organ Indexes in NOD Mice

The organ indexes in NOD mice are shown in Table 4. Compared with that of group NC mice and group MC mice, the indexes of spleen and liver in group FTY720 mice were significantly lower (*P* < 0.01 or *P* < 0.05), which demonstrated that the spleen and liver of NOD mice had shriveled in the progression of administration with FTY720. It was speculated that FTY720 is toxic to the spleen and liver of NOD mice. Compared with group NC mice and MC, the indexes of thymus, heart and lung of group FTY720 were not significantly different. Compared with group NC, the brain of group FTY720 and MC was significantly greater, while the one of thymus of group MC was very significantly less.

### 2.5. The Body Weight of NOD Mice

Before the trial, the average body weight of mice in the group NC, group MC and FTY720-treated group were 28.77 g, 21.52 g and 21.65 g respectively (shown in Table 5). By the end of the trial, the mice in group NC exhibited an average weight gain of ~7.4 g (~26%), while the mice in group MC and group FTY720 both exhibited a slight weight gain of 2–3 g (13.3% and 10.3% respectively). The body weight of group FTY720 mice was slightly lower than that of group MC, which was only significantly different in the 4th and 7th weeks of the trial (*P* < 0.01 or *P* < 0.05). These two groups both had less body weight than group NC (*P* < 0.01).

## 3. Discussion

The non-obese diabetic (NOD) mouse is a useful experimental tool that can help improve the understanding of disease pathophysiology as T1D animal models. In this study, we confirmed that Type 1 diabetes in NOD mice could be suppressed effectively by FTY720. This result is consistent with previous findings [7]. Glutamic acid decarboxylase (GAD65) is one of the first cell antigens and the strongest incentives to provoke autoimmune responses in both humans and the NOD mouse [11,13]. The presence of islet-related autoantibody to β-cell, including insulin, glutamic acid decarboxylase (GAD, isoforms GAD65 and GAD67) were provided not only in animal models such as NOD mice, but also in the preclinical period of the disease in humans [10,11,14]. The presence of GAD autoantibodies has been shown to be a strong predictive marker for the eventual onset of T1D [14]. The assay of anti-GAD antibody demonstrated that FTY720 treatment evidently suppressed the anti-GAD response. Some researchers concluded that FTY720 suppresses *in vivo* immune functions mainly by inducing systemic lymphopenia to inhibit T cell functions in normal mice [15,16]. The studies have been carried out in NOD mice or biobreeding rats treated with a single dose of FTY720 (0.5 mg/kg, 1 mg/kg/d or 10 mg/kg) to search for potential mechanisms of immunosuppression on Type 1 diabetes [2,8,9]. The 1 mg/kg dose widely adopted was based on the recommendation by Novartis as a dose that causes lymphopenia by sequestration and not by apoptosis [17].

## 4. Experimental Section

### 4.1. Materials

FTY720, a white crystalline powder (HPLC purity: 99.9%) were purchased from Hangzhou Tongming Pharmaceuticals. Female non-obese diabetic (NOD) mice and ICR mice (Production License: SCXK (Shanghai) 2007–0005) which were of SPF grade were purchased from Shanghai Laboratory Animal Center, Chinese Academy of Sciences (SLAC, CAS, Shanghai, China) Glutamic acid decarboxylase autoantibody (GAD-Ab) ELISA Kit was purchased from Beijing Limi Biotechnology (Beijing, China). All other reagents were of analytical grade unless otherwise specified.

### 4.2. NOD Mice

Twenty, 12-week-old female NOD mice and 10 ICR female mice (Use License: SYXK (Zhejiang) 2003–0003) were selected for these experiments. The animals were housed in an SPF animal laboratory of barrier system at 22 ± 1 °C, with a 12 h light-dark cycle, the humidity of 50–70% and low noise (<50 dB). The animals were fed a complete diet. All mice were allowed free access to food and filtered water.

### 4.3. NOD Mice Grouped and Treated by FTY720

The 12-week-old female NOD mice, which weighed 19–25 g and had no diabetic symptoms, according to blood glucose levels and body weight, were randomly divided into two groups: a FTY720-treated group (*n* = 10), which were fed 1 mg·kg^−1^ of FTY720 daily (group FTY720) [8], and a model control group (*n* = 10), which were fed 10 mL·kg^−1^ of physiological saline daily (group MC). In addition, 10 female ICR mice considered as normal control group (group NC) were fed 10 mL·kg^−1^ of physiological saline daily. The experimental treatments were in agreement with the Guide for the Care and Use of Laboratory Animals issued by the National Institute of Health.

### 4.4. Assessment of Diabetic Symptoms

At the age of 12 weeks, each mouse was fed with 0.1 mL/10 g once a day for 10 successive weeks. The mice were weighed and blood was taken from the tail vein to determine fasting blood glucose values using a OneTouch^®^ Ultra glucometer (Johnson, Buren Zwick, NJ, USA), once a week. Diabetes was determined when a blood glucose level exceeded 10.0 mmol/L on two consecutive weekly tests. At 24 h after the last administration, blood was taken from mice and serum was separated by centrifugation (3000 rpm, 10 min).

The anti-GAD antibody was assayed using an enzyme-linked immunosorbent assay (ELISA) method. The plate wells were prepared for diluted standard, blank and samples. 100 μL of dilutions of standard were added into each well, and then covered with the plate sealer and incubated for 2 h at 37 °C. After removing the liquid of each well, 100 μL of Detection Reagent A working solution was added to each well and incubated for 1 h at 37 °C after covering it with the plate sealer. Following aspiration of the solution, the wells were washed with 400 μL of wash solution. 100 μL of Detection Reagent B working solution were added to each well and incubated for 30 min at 37 °C after covering it with the plate sealer. The aspiration/wash process was repeated five times. 90 μL of Substrate Solution was added to each well and covered with a new plate sealer. After being incubated for 15 min at 37 °C, 50 μL of stop solution was added to each well. Measurements were conducted on the plate at 450 nm on the microplate reader immediately after the removal of any water drops.

Observation ended at the age of 22 weeks. Then the mice were sacrificed. The spleen, thymus, kidney, liver and heart were harvested and weighed for their organ indexes.

### 4.5. Statistical Analysis

With the exception of the data of cumulative incidence, all other data were expressed as means ± standard deviation (SD). Comparisons of cumulative incidence of diabetes between treated and the model control group was analyzed using the *x^2^* test, and other comparisons between groups were analyzed by one-way ANOVA. All statistical methods were performed by the statistical software Statistical Package for Social Sciences 13.0 (SPSS: Chicago, IL, USA, 2001). Values of *P* < 0.05 and *P* < 0.01 were considered to be significant and very significant respectively.

## 5. Conclusions

We investigated the physiological and biochemical aspects under FTY720 treatment of the dose of 1 mg/kg/d in our current research stage to see if there are new discoveries, which is hoped to complement this area. A previous study reported that FTY720 enables rat spleen cells to kill cells showing features of apoptosis [18]. The major study showed that FTY720 may be an effective agent for the treatment of type 1 diabetes [1,2,8,9,19,20]. According to the indexes of metabolic organs in this research, the question of whether FTY720 is toxic to the spleen, kidney or liver needs further investigation. The future work will be carried out in the area of dose effect of FTY720 on NOD and its possible toxicity.

## Figures and Tables

**Table 1:** Effect of FTY720 on blood glucose in NOD mice (mmol/L, X̄ ± s, *n* = 10).

Group/Dosage		NC (10 mL·kg^−1^)	MC (10 mL·kg^−1^)	FTY720 (1 mg·kg^−1^)
Before Administration		5.03 ± 1.08	4.02 ± 0.53 ^ΔΔ^	3.91 ± 0.72 ^ΔΔ^
After Administration	1	5.45 ± 0.76	4.90 ± 1.06	3.86 ± 0.46 ^ΔΔ^
	2	4.85 ± 0.81	3.90 ± 0.69 ^Δ^	4.11 ± 0.55 ^Δ^
	3	6.51 ± 0.44	9.24 ± 7.59	5.50 ± 0.70 ^ΔΔ^
	4	5.87 ± 0.50	11.60 ± 10.31	5.27 ± 0.73 ^Δ^
	5	5.75 ± 0.49	12.14 ± 10.26	5.41 ± 0.66 ^*^
	6	6.58 ± 0.45	13.45 ± 10.74	6.00 ± 0.55 ^*,Δ^
	7	6.73 ± 0.54	13.05 ± 9.35 ^Δ^	5.61 ± 0.33 ^*,ΔΔ^
	8	6.30 ± 0.59	16.42 ± 11.51 ^Δ^	5.94 ± 0.75 ^**^
	9	6.62 ± 0.46	18.76 ± 11.12 ^ΔΔ^	7.03 ± 0.76 ^**^
	10	7.13 ± 0.66	18.92 ± 11.76 ^Δ^	5.76 ± 0.62 ^**,ΔΔ^

Note: Compared with normal control, ^Δ^ *P* < 0.05; ^ΔΔ^ *P* < 0.01;

Compared with model control, ^*^ *P* < 0.05, ^**^ *P* < 0.01.

**Table 2:** Effect of FTY720 on the incidence of diabetes in NOD mice (*n* = 10).

Group/Dosage		NC (10 mL·kg^−1^)	MC (10 mL·kg^−1^)	FTY720 (1 mg·kg^−1^)
Before Administration		0	0	0
After Administration	1	0	0	0
	2	0	0	0
	3	0	2	0
	4	0	2	0
	5	0	3	0 ^*^
	6	0	4	0 ^*^
	7	0	5	0 ^**^
	8	0	6	0 ^**^
	9	0	7	0 ^**^
	10	0	7	0 ^**^

Note: Compared with normal control, ^Δ^ *P* < 0.05; ^ΔΔ^ *P* < 0.01.

Compared with model control, ^*^ *P* < 0.05; ^**^ *P* < 0.01.

**Table 3:** Effect of FTY720 on Optical Density (OD) value of anti-GAD antibody in NOD mice (*n* = 10).

Group/Dosage	NC (10 mL·kg^−1^)	MC (10 mL·kg^−1^)	FTY720 (1 mg·kg^−1^)
OD value	0.429 ± 0.132	1.557 ± 0.176 ^ΔΔ^	0.798 ± 0.369 ^**,Δ^

Note: Compared with normal control, ^Δ^ *P* < 0.05; ^ΔΔ^ *P* < 0.01.

Compared with model control, ^*^ *P* < 0.05; ^**^ *P* < 0.01.

**Table 4:** Effect of FTY720 on organ indexes in NOD mice (g/kg, X̄ ± s, *n* = 10).

Group/Dosage	NC (10 mL·kg^−1^)	MC (10 mL·kg^−1^)	FTY720 (1 mg·kg^−1^)
Thymus	2.386 ± 0.870	1.493 ± 0.223 ^ΔΔ^	1.768 ± 0.476
Spleen	3.957 ± 0.643	3.932 ± 0.338	2.884 ± 0.422 ^**,ΔΔ^
Kidney	11.80 ± 2.23	12.83 ± 1.93	11.20 ± 1.09 ^*^
Liver	42.09 ± 3.22	50.80 ± 15.00	36.00 ± 2.85 ^*,ΔΔ^
Heart	4.84 ± 0.47	4.84 ± 1.00	4.73 ± 0.57
Brain	13.52 ± 0.98	20.82 ± 0.98 ^ΔΔ^	20.30 ± 4.77 ^ΔΔ^
Lung	6.67 ± 1.01	7.54 ± 0.86	7.28 ± 0.64

Note: Compared with normal control, ^Δ^ *P* < 0.05; ^ΔΔ^ *P* < 0.01.

Compared with model control, ^*^ *P* < 0.05; ^**^ *P* < 0.01.

**Table 5:** Effect of FTY720 on body weight in NOD mice (g, X̄ ± s, *n* = 10).

Group/Dosage		NC(10 mL·kg^−1^)	MC (10 mL·kg^−1^)	FTY720 (1 mg·kg^−1^)
Before Administration		28.77 ± 1.96	21.52 ± 1.78 ^ΔΔ^	21.65 ± 1.88 ^ΔΔ^
After Administration	1	30.78 ± 1.94	22.70 ± 1.21 ^ΔΔ^	21.36 ± 1.49 ^ΔΔ^
	2	31.07 ± 2.41	22.60 ± 1.34 ^ΔΔ^	21.76 ± 1.24 ^ΔΔ^
	3	33.15 ± 2.46	22.99 ± 1.55 ^ΔΔ^	22.41 ± 1.46 ^ΔΔ^
	4	34.23 ± 2.87	23.72 ± 1.59 ^ΔΔ^	22.38 ± 1.14 ^*,ΔΔ^
	5	34.85 ± 3.27	23.58 ± 2.12 ^ΔΔ^	22.31 ± 1.50 ^ΔΔ^
	6	36.19 ± 3.43	24.46 ± 1.73 ^ΔΔ^	23.34 ± 1.69 ^ΔΔ^
	7	35.25 ± 3.04	24.75 ± 1.20 ^ΔΔ^	22.33 ± 1.20 ^**,ΔΔ^
	8	35.63 ± 2.87	24.55 ± 1.11 ^ΔΔ^	23.33 ± 1.96 ^ΔΔ^
	9	35.65 ± 2.85	26.65 ± 2.89 ^ΔΔ^	23.47 ± 1.45 ^ΔΔ^
	10	36.17 ± 2.84	24.38 ± 2.73 ^ΔΔ^	23.88 ± 1.49 ^ΔΔ^

Note: Compared with normal control, ^Δ^ *P* < 0.05; ^ΔΔ^ *P* < 0.01.

Compared with model control, ^*^ *P* < 0.05; ^**^ *P* < 0.01.
